# Supplementary material for: A comparison of dynamic warm-up and “warm-up” using self-massage tools on subsequent sit-and-reach displacement
Source: PLoS One. 2024 Aug 22;19(8):e0307073. doi: 10.1371/journal.pone.0307073 (PMC11341030; doi:10.1371/journal.pone.0307073)
Supplement: Attachment — Submitted filename: my comments for PLOS journal.docx [file pone.0307073.s003.docx]

The manuscript entitled (” No difference between dynamic warmup and “warmup” using self-massage tools on subsequent sit-and-reach performance “) has compared different approaches as warm-up protocols. However, there are some comments as follows.

1. Title: It’s better to change the title as shows comparing methods not the findings of the manuscript.
2. Abstract: Objectives: lines 3, 4: correct the acronym DWU after the word leg cycling. You can use this acronym in the first line of the objectives section.
3. Keywords: In my opinion, “range of motion” is not suitable because indeed, you hadn’t measured ROM and ROM is more related to joints. Also, it’s better to use warm up (or warm-up) instead of warmup especially since you have used the acronym WU not W.
4. In this way, in the abstract conclusion it’s better to say: displacement in sit and reach test.
5. Introduction: You have stated “ We hypothesized that both FR and PM would result in similar (with respect to both magnitude and time course) sit-and-reach acute changes in performance as DWU”, so why you have compared their effectiveness?
6. Please explain what was the rationale for choosing the sit and reach test as the only outcome measure. You targeted different muscle groups including hamstrings, gastrocnemius, gluteus maximus, and lumbar extensors. Indeed this test is more suitable for measuring hamstring flexibility and plantar flexors are not as important as hamstrings.
7. How did you calculate the sample size?
8. In the foam rolling section: add the unit of dimensions.
9. In percussive massage, add the reason for moving from proximal to distal of the limb/area.
10. Mention the order of muscles targeted in FR and PM.
11. Add a section about “the assessment” and explain the details for measuring the score of sit and reach test as your only outcome measure. Also, I recommend adding a figure to show the measuring. How did you convert the score to the percent and so on?
12. Mention the name of the company and country of SPSS software.
13. Did you assess the hamstring length of the participants before being included in the study?
14. Table 1: add the full form of PRE below the table.
15. Please add effect size for each intervention result. It could help to better compare at least within groups.
16. I suggest adding one graph for changes in scores SRT between and within groups. It is more useful than the figure 2. You can present Figure 2 results in a separate table. Also, you can use MANCOVA analysis to omit the baseline difference in the FR group. What is your opinion about this difference?
17. Discussion: It seems that the first group's muscle tissue temperature may be returned to the baseline value before starting the intervention. I suggest presenting your findings also based on different muscle groups at least for FR and PM.
18. Conclusion: you stated “ we suggest that FR and PM are viable alternate “warm-up” strategies when the objective is to acutely increase range of motion. “. Based on my previous suggestions, it should be changed.
